# A Descriptive Study of Patients Presenting With Topical Steroid Damaged/Dependent Face (TSDF) to a Tertiary Care Center in Northern India

**DOI:** 10.7759/cureus.75566

**Published:** 2024-12-11

**Authors:** Satyendra K Sharma, Snigdha Meher, Nikhil Ranjan Das, Siddhartha Dash

**Affiliations:** 1 Dermatology, Venereology and Leprosy, Hind Institute of Medical Sciences, Sitapur, IND; 2 Dermatology, Hind Institute of Medical Sciences, Sitapur, IND; 3 Dermatology, Venereology and Leprology, Saheed Laxman Naik Medical College, Koraput, IND; 4 Dermatology and Venereology, Srirama Chandra Bhanja (SCB) Medical College and Hospital, Cuttack, IND

**Keywords:** acneiform eruptions, facial dermatoses, steroid induced rosacea, topical corticosteroid, topical corticosteroid damaged/dependent face

## Abstract

Introduction

Topical steroid damaged/dependent face (TSDF) is defined as the semi-permanent or permanent damage to the skin of the face precipitated by the irrational, indiscriminate, or prolonged use of topical corticosteroids (TCs), resulting in various cutaneous signs and symptoms and psychological dependence on the drug. The objective was to determine the clinical spectrum of TSDF.

Methods

This was an observational cross-sectional study conducted between May 2021 and April 2022, comprising 100 consecutive patients of TC-induced facial dermatoses who visited the skin and venereal disease OPD of a tertiary care hospital in northern India. Any case with a negative history of TC abuse was excluded from the study.

Result

The study included 100 subjects of TSDF. Females outnumbered males and the age of the patients ranged from 12 to 55 years with a mean age of 27.6 ± 10.07 years. The majority of patients used TCs on the face for pre-existing acne (41%), followed by melasma (20%). Over-the-counter (63%) was the most common method of acquiring TCs, followed by prescriptions from non-qualified persons (17%) and those provided by friends and relatives (9%). Mometasone (41%) was found to be the most commonly misused TC, followed by clobetasol (31%), and betamethasone (29%). The most common morphological presentation was erythema (42%), followed by acneiform eruptions (22%), steroid-induced rosacea (21%), hyperpigmentation (20%), hypertrichosis (5%) and perioral dermatitis (3%).

Conclusion

This study highlights the impact of misuse of TCs on the face in a single-center setting and provides a detailed description of the associated factors. Such studies could play a crucial role in addressing this issue. Moreover, strict enforcement of regulations on pharmaceutical companies and non-qualified individuals prescribing TCs could help in decreasing this growing health hazard.

## Introduction

Topical corticosteroids (TCs) were first introduced by Sulzberger and Witten in 1952, revolutionizing the treatment of dermatological disorders, particularly inflammatory dermatoses [1]. TCs have an anti-inflammatory, antiproliferative, immunosuppressive, anti-pruritic, atrophogenic, melanopenic, and sex-hormone-like effect on the skin. To our knowledge, TC misuse on the face was first reported in India in 2006 [2]. Topical steroid damaged/dependent face (TSDF) is defined as the semi-permanent or permanent damage to the skin of the face precipitated by the irrational, indiscriminate, unsupervised, or prolonged use of TCs resulting in various cutaneous signs and symptoms and psychological dependence on the drug [2]. These signs and symptoms range from erythema, monomorphic acne, atrophy, telangiectasia, rosacea, perioral dermatitis, striae, hypertrichosis, and demodicosis, with sudden cessation causing rebound erythema, burning and scaling leading to further TC dependence among patients [2,3].

The facial skin is thinner compared to the skin on other body parts which makes it more vulnerable to ill effects of environmental factors as well as drugs or cosmetics. Less potent TCs should be applied on the facial skin for not more than two weeks [2]. TC misuse on the face has reached alarming proportions in India due to various factors like cheap and easily available over-the-counter (OTC) combination creams containing TCs; unethical and uninformed prescription of these preparations to patients by non-dermatologists, alternative medicine practitioners, and quacks; unauthorized selling of these preparations by chemists; desire for fairer skin among youth leading to unauthorized use of depigmenting preparations such as Kligman’s regimen; and apathetic government agencies neglecting these issues [2].

Though TSDF has been studied extensively in the world, including Asia and India, we want to highlight the impact of this condition to the community in this part of the country and assess the clinical presentations and factors associated with the misuse of TCs.

## Materials and methods

Study overview

This observational, cross-sectional study was conducted between May 2021 and April 2022, involving 100 consecutive patients who visited the skin and venereal disease OPD of Hind institute of medical sciences, Sitapur and were diagnosed with TC-induced facial dermatoses.

Institutional ethics committee approval was obtained before the commencement of the study (HIMS/IRB/2020-21/82).

Inclusion/Exclusion Criteria

All patients of both sexes with complaints of facial dermatoses with a positive history of TC application and who were 10 years of age or above were included in this study. Those who were unwilling to give consent and those who had comorbidities that resembled/could cause changes similar to TC side effects (e.g., polycystic ovaries/Cushing's syndrome/thyroid disorders) were excluded from the study. Minors <10 years of age and any case with a negative history of TC abuse were also excluded from the study. 

Study Procedure and Assessments

After obtaining informed consent, detailed history was taken including age, gender, education, the potency of TCs used, prescriber of TCs, the disease for which TC was used, signs and symptoms for which the patient presented, duration of the disease, and the duration for which TC was used over the face. The relevant details of the patient, examination findings, investigations, and diagnosis were recorded in the standard proforma.

Statistical analysis

The data collected were entered into a Microsoft Excel worksheet (Microsoft Corp., Redmond, WA). Descriptive statistics were computed. Mean and standard deviation were computed for continuous variables. Frequency and proportions were computed for categorical variables.

## Results

A total of 100 consecutive patients having TC-induced facial dermatoses were included in the study. The age of the patients ranged from 12-55 years with a mean age of 27.6 ± 10.07 years. The majority of our patients belonged to the age group of 11-30 years (n=71, 71%). Female patients constituted the majority of our study population (n=72, 72%). Most participants belonged to a rural background (n=78, 78%). The patients used TCs on the face because of pre-existing conditions such as acne (n=41, 41%), followed by melasma (n=20, 20%). The third most common indication for using TCs was to achieve a fairer complexion, accounting for 12% of cases (n=12). Other conditions for which TCs were used on the face included facial itching, tinea faciei, casual routine use as a day care cream, polymorphic light eruption, and urticaria. Most of the patients used TCs on the face as an OTC drug (n=63, 63%). For the rest of the patients, it was prescribed by non-qualified persons (n=17, 17%), friends and relatives of patients (n=9, 9%), general practitioners (n=4, 4%) and dermatologists (n=3, 3%). Four patients used TC on their faces after viewing advertisements on social media as well as to finish unused medications at home without consultation. Most of the patients used TCs of varying potencies, ranging from low to super potent. The most commonly used preparations were mometasone (n=41, 41%), clobetasol (n=31, 31%), and betamethasone (n=29, 29%) (Table 1).

**Table 1:** Demographic characteristics of the study population

Demographic characteristics		N (%)
Sex	Male	28 (28%)
	Female	72 (72%)
Locality	Rural	78 (78%)
	Urban	22 (22%)
Age group (in years)	11-20	23 (23%)
	21-30	48 (48%)
	31-40	18 (18%)
	41-50	9 (9%)
	>50	2 (2%)
Prescriber	Over the counter	63 (63%)
	Non-qualified person	17 (17%)
	Friend and relative	9 (9%)
	General practitioner	4 (4%)
	Dermatologist	3 (3%)
Nature of steroid	Mometasone	41 (41%)
	Clobetasol	31 (31%)
	Betamethasone	29 (29%)
	Beclomethasone	2 (2%)

The duration of presenting symptoms ranged from one to six months (n=46, 46%). It was observed that 39% of patients had symptoms for less than a month whereas 15% of patients had disease manifestation for more than six months. Redness over the face was the most common symptom present in (n=42, 42%) of subjects. Furthermore, a burning sensation was mentioned in 38% of cases, itching in 26%, elevated reddish papules over the face in 22%, unwanted facial hair growth in 3%, and facial swelling in 2%.

**Figure 1:**
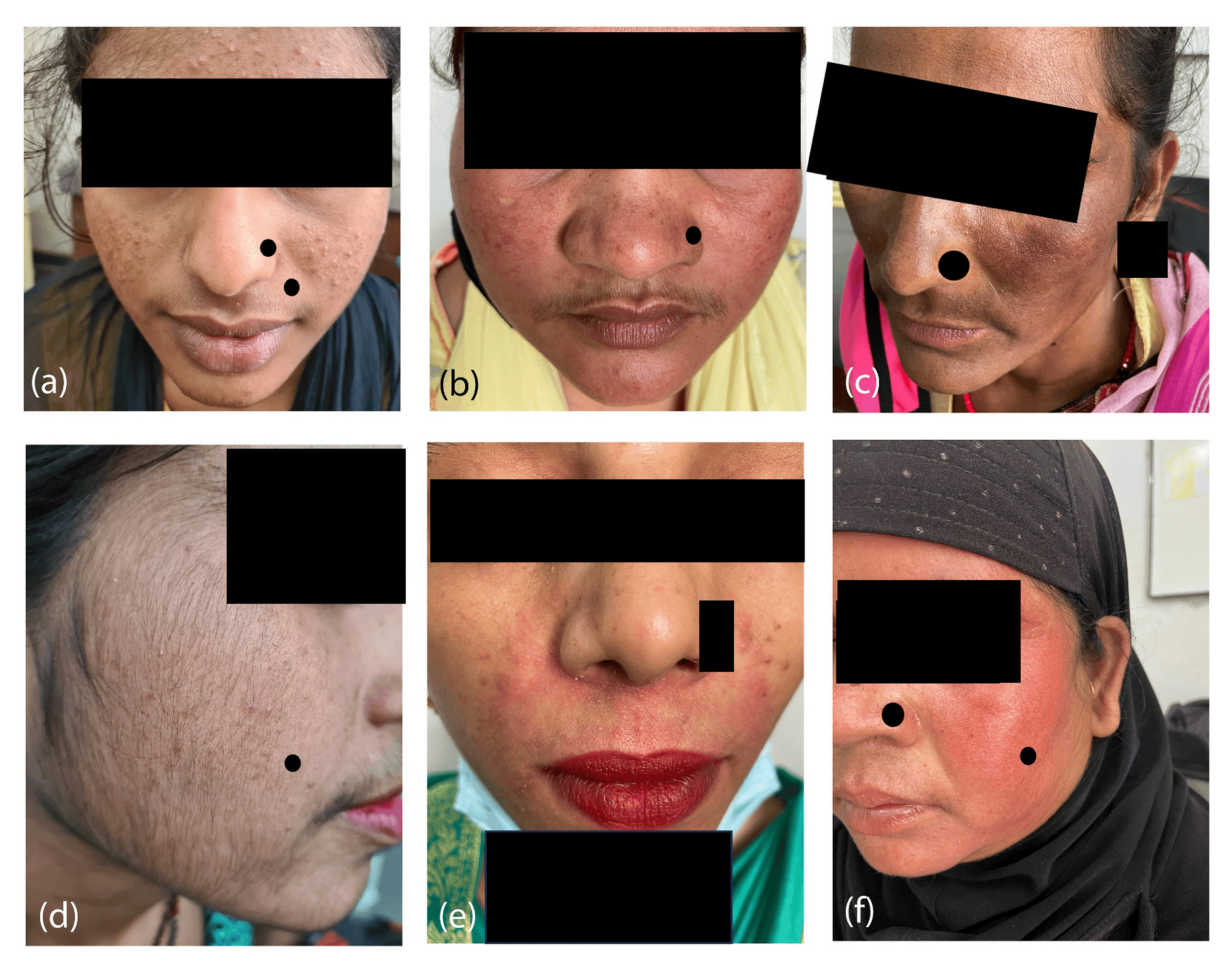
Morphological presentation of topical steroid-damaged face a. Acneiform lesion (22/F); b. Erythema (19/F); c. Hyperpigmentation (26/F); d. Hypertrichosis (18/F), e. Perioral dermatitis (28/F), f. Steroid-induced rosacea (32/F).

Our study population exhibited a variety of morphological presentations. The most common morphological presentation was erythema (n=42, 42%) (Figure 1b), followed by acneiform eruptions (n=22, 22%) (Figure 1a), steroid-induced rosacea (n=21, 21%) and hyperpigmentation (n=20, 20%) (Figure 1c). Furthermore, a combination of presentations was seen such as acneiform lesions with hyperpigmentation (n=2, 2%). Hypertrichosis (Figure 1d) and steroid-induced perioral dermatitis (Figure 1e) were found in 5% and 3% of cases, respectively (Table 2).

**Table 2:** Clinical characteristics of the study population

Clinical characteristics		N (%)
Duration of symptoms (in month)	<1	39 (39%)
	1-6	46 (46%)
	>6	15 (15%)
Adverse effects	Erythema	42 (42%)
	Acneiform lesion	22 (22%)
	Steroid-induced rosacea	21 (21%)
	Hyperpigmentation	20 (20%)
	Hypertrichosis	5 (5%)
	Perioral dermatitis	3 (3%)

## Discussion

In our study, there was a wide variation in age distribution ranging from 12 to 55 years with a mean age of 27.6 years. The majority of patients (71%) belonged to the age group of 11-30 years. Similar findings were seen in a study by Jain et al. conducted among an Eastern Indian population, where the mean age of female and male patients were found to be 25.6 years and 23.8 years respectively, and most of the patients were between the age group of 20-30 years [4]. In a study conducted among a North Indian population, Sharma et al. found 85% of the study population to be between 21 and 50 years of age [5]. Al-Dhalimi et al.'s study on an Iraqi population found that 76.4% of the study population were 10-29 years of age, which is similar to our findings [6].

Female patients constituted the majority (72%) of our study population. In studies conducted by Jain et al. and Lu et al., 83.22% and 91.9% of the study populations were female, respectively [4,7]. The relatively higher proportion of men in our study (28%) could be attributed to their increasing desire for blemish-free and fairer complexions, which leads to the misuse of TCs on the face. In our study, 78% of patients belonged to rural backgrounds. Jain et al. found similar findings with 68.7% of patients belonging to rural backgrounds [4]. A study by Saraswat et al. found that 54% of patients belonged to urban areas [3]. This finding differs from our study and may be due to the bias that ours is a hospital-based study which is present in a rural locality.

In the study done by Pal et al. in an Eastern Indian population, the most common dermatoses for which TCs were used on the face was acne (32.47%), followed by use as a fairness cream (19.11%) [8]. In the study by Sharma et al., 36% of patients used TCs on the face for fairer skin, followed by 29.5% of patients with acne-related concerns [5]. Al-Dhalimi et al. found that 65.7% of the study population used TCs as skin-lightening cream, followed by 16.4% of patients using the same for acne [6]. In our study, the most common indications for the use of TCs on the face were acne (41%), melasma (20%), and depigmentation of the skin (12%). This suggests that the widespread desire for fairer and clearer skin may lead individuals to misuse TCs. Prevailing illiteracy among the population, advertisements glorifying fairer skin, and unchecked prescription of TCs by non-specialists contribute to this burden [3]. Mukhtar et al. found that lighter skin color gives women more confidence, better job opportunities, and better chances of getting married, and also found that advertisements on television glorifying lighter skin color influence their preference for having lighter skin tones [9].

Most of our patients (63%) used TCs on the face as OTC drugs. Other most common advisers were non-qualified persons and friends and relatives. In studies conducted by by Pal et al., Sharma et al., and Jha et al., OTC use of TCs was the most common mode by which patients got treatment i.e., 39.85%, 34.5%, and 42.9%, respectively [5,8,10]. In the study by Al-Dhalimi et al., it was found that most patients obtained TCs through paramedical personnel as well as family, friends, and self-use [6]. Rathi et al. found the most common providers of TCs were friends, chemists, beauticians, and relatives [11]. These findings indicate the lack of regulation by authorities on the sale of these drugs without prescription, patients misled by pharmaceutical companies for the sale of their products by commercials and advertisements without highlighting the risks of misuse, lack of fear among non-qualified persons in prescribing TCs to patients due to absence of check on them by authorities and lack of awareness among patients and their friends and family members about the risk of misuse of TCs [2].

In our study, most patients presented with symptoms of one to six months duration. Our findings were similar to those of Pal et al. [8] whereas other studies found the most common duration of symptoms to be around one year [4,6,12-14]. The most common presentation in our study was erythema (42%), followed by an almost equal distribution of acneiform eruption, rosacea (Figure 1f), and hyperpigmentation. These findings are different from other studies, which found acne and acneiform eruption to be the most common side effects [3,4,6,12-18]. However, Pal et al. found rosacea/photosensitivity to be the most common side effect [8]. Seandrosoa et al. in their study of patients from the region of Madagascar found pigmentation disorders and cutaneous atrophy to be the most common adverse effects [19].

The limitation of this study was the small sample size. There may have been an underestimation in the detection of TSDF cases and its clinical profile, as many patients found it difficult to recall the frequency and duration of application as well as the generic and brand names of topical corticosteroids or triple combination creams, due to the wide availability of these products. The diagnosis was mostly clinical and confirmed by their respective investigations. The frequency of demodicosis leading to these findings was not estimated. Dermoscopic evaluations of the lesions were not done. Another limitation was the non-assessment of systemic adverse effects due to the use of TCs.

Regulations by concerned authorities to restrict the over-the-counter sale of TCs along with strict punishment for pharmaceutical companies who spread advertisements among the community without addressing the risks associated with TCs for their commercial benefit could go a long way in preventing TSDF. Dermatologists and other healthcare professionals should raise awareness about the potential hazards of TCs among the public. Quacks who prescribe TCs to patients without proper qualifications should be stopped and prosecuted.

## Conclusions

In the present study, we found that TCs were primarily obtained over the counter, followed by prescription by non-qualified persons, and friends and relatives. This indicates that TSDF is a growing healthcare concern and could reach epidemic proportions if left unchecked. Therefore, we suggest that regulatory bodies work to restrict the over-the-counter distribution of TCs and to punish unauthorized prescriptions of these medications by non-qualified persons; additionally, healthcare authorities should also spread awareness regarding the hazards of such medications.
